# A pH-Dependent Phase Separation Drives Polyamine-Mediated Silicification from Undersaturated Solutions

**DOI:** 10.1021/acsnano.4c08707

**Published:** 2024-12-06

**Authors:** Protap Biswas, Nitzan Livni, Debojit Paul, Lior Aram, Razi Safadi, Neta Varsano, Nadav Elad, Roman Kamyshinsky, Michal Leskes, Assaf Gal

**Affiliations:** a Department of Plant and Environmental Sciences, Weizmann Institute of Science, Rehovot 7610001, Israel; b Department of Molecular Chemistry and Materials Science, Weizmann Institute of Science, Rehovot 7610001, Israel; c Department of Chemical Research Support, Weizmann Institute of Science, Rehovot 7610001, Israel

**Keywords:** biosilicification, phase separation, silica synthesis, sol−gel process, macromolecular condensates

## Abstract

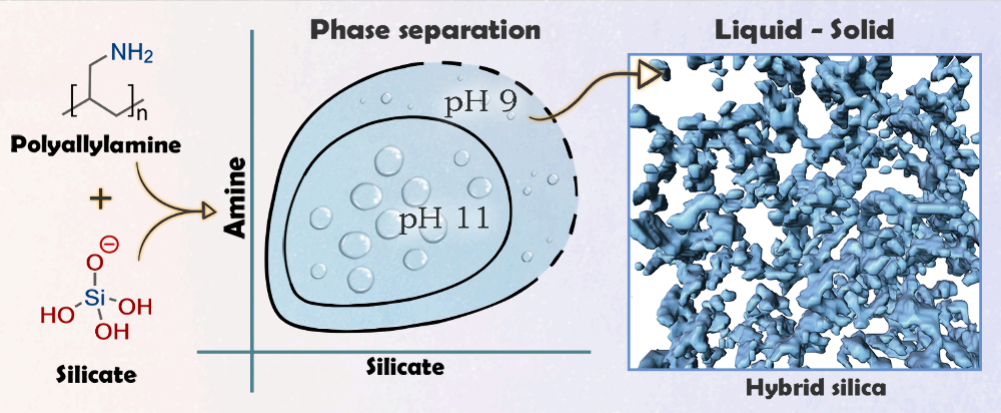

Silica polymerization from its soluble monomers is fundamental to many chemical processes. Although industrial methods require harsh conditions and concentrated precursors, biological silica precipitation occurs under ambient conditions from dilute solutions. The hallmark of biosilica is the presence of amine-rich organic macromolecules, but their functional role remains elusive. Here, we show a pH-dependent stimulatory effect of such polyamines on silica polymerization. Notably, this process is decoupled from the saturation degree, allowing the synthesis of polymer–silica hybrid products with controlled network morphologies from undersaturated solutions. The data suggest a two-step phase separation process. First, an associative liquid–liquid phase separation forms a micrometer-size dense phase. Second, silica undergoes a liquid-to-solid transition in the supersaturated condensates to form a bicontinuous silica structure. This study can inspire “soft chemistry” routes to design organic–inorganic nanomaterials with regulatory principles optimized by evolution.

Silica is one of the most versatile and abundant materials in nanotechnological applications. Traditional synthesis procedures of silica from monomeric silicate species require harsh chemical conditions for silica polymerization.^1,2^ Nevertheless, the development of aqueous sol–gel routes for silica production at physiological conditions opened new avenues for silicification using “soft” chemistry.^3,4^ Developments in such biocompatible routes drive constant inspiration from the living world, in which several organisms, such as the unicellular algae diatoms, are producing functional silica structures from the dilute seawater environment in which they live.^5−7^ One of the blueprints of diatom silica is the inclusion of a unique class of biomacromolecules, long-chain polyamines (LCPA).^8^ Therefore, many bioinspired studies investigated how various polyamines can affect silicification under physiological conditions.^9,10^

To date, the reports on the effects of polyamines on silica polymerization do not provide a clear consensus.^9^ Some works conclude that polyamines stabilize silica monomers from polymerization, while others found that the polymer promotes silicification.^11−16^ These apparent contradictions can originate from different experimental setups. For example, the silica sources vary from sodium silicate solution (“glass water”) to organic ethoxysilanes, the polymers differ in their functional groups, chain length, chemical structure, and reaction conditions such as concentration, pH, and salt content are also case specific.^10^ Therefore, it is clear that a necessity for further advancement in the field is a better chemical understanding of the functional interactions between silica monomers and polyamines.

Many of the previous works concentrated on mildly acidic pH values. The concept of acidic pH conditions arises from the fact that silica is most insoluble at this pH range and indirect evidence suggests that this is the relevant pH for diatom silicification.^17^ Since the solutions used in this pH range are supersaturated, the polymerization of silicate monomers is thermodynamically driven and it was postulated that the polyamines act as catalysts that reduce kinetic barriers to favor precipitation via the sol–gel silica pathway.^1,3^ However, such direct catalytic activity is not straightforward because of the scarcity of polymer molecules in biogenic silica that precludes direct interaction with all silanol bonds.^18^ In addition, the synthetic systems usually contain additional components such as phosphate buffers, peptides, and other polymers.^6,9^ These compositions evoke several hypotheses for alternative chemical processes in which polyamines are involved in biosilicification. One of them includes phase separation as an important step in biosilicification.

The idea of phase separation during biosilicification arises from the involvement of the polycationic polyamines and negatively charged proteins embedded in diatom silica.^19,20^ It was shown in vitro that phase separation of surfactants,^21^ electrolytes,^22,23^ or polyamine-rich phases induces the precipitation of silica.^24^ Nevertheless, since phase separation was proposed to occur at various scenarios,^25^ for example, electrostatically between polymers,^22,26^ between polymers and inorganic ions,^27^ and between silica species, it is unclear what is the relevant phase separation reaction that is fundamental for polymer-mediated silicification.^28^ Taken together, even though many works have used polymer-mediated silicification, the underlying chemical mechanism by which polyamines and soluble silica interact is unknown.

In this work, we set to elucidate the interactions between a simple synthetic polyamine, poly(allylamine hydrochloride) (PAH.HCl) and the soluble silica in glass water. We tested a wide concentration range of polymer, silica, and pH values to monitor the effects of the polymer on silica. We show that pH is a pivotal driver of the chemical interactions, as at high pH values, an associative phase separation between charged silica species and neutral polymer chains results in polymerization of a silica–polymer hybrid material in undersaturated conditions. Such a two-step process, which involves liquid–liquid and liquid-to-solid phase separation processes,^29−31^ is known in biological condensates and allows fine-tuned control over the resulting products.

## Results and Discussion

**Figure 1:**
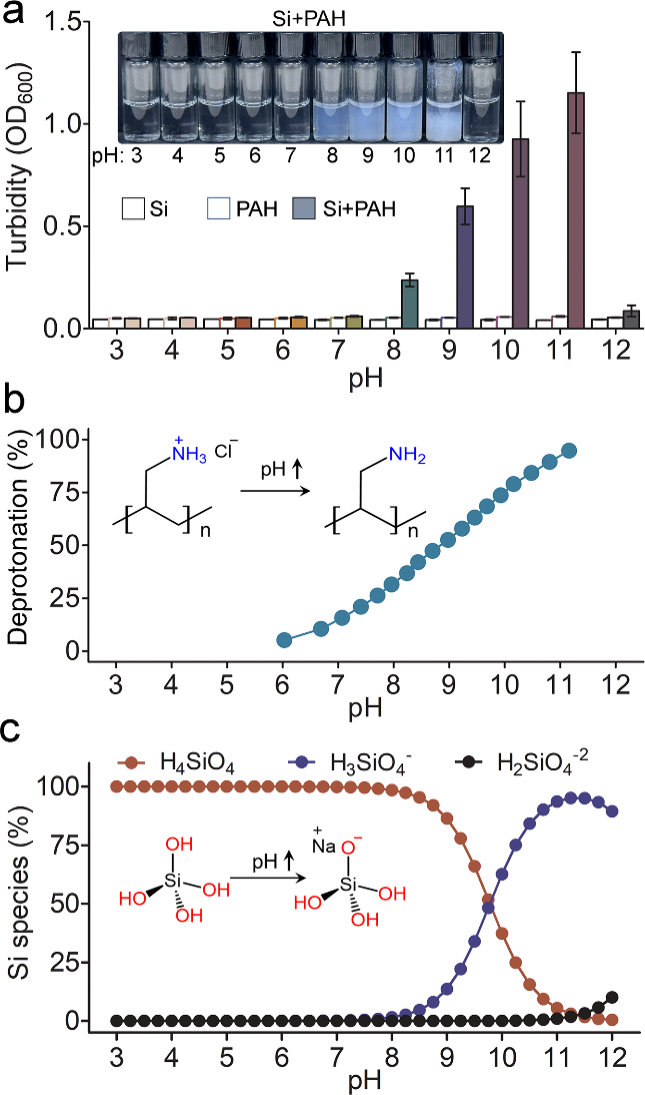
Effect of PAH on silica precipitation as a function of pH. (a) Turbidity measurements of the different solutions: Si-50 mM silicate, PAH-50 mM polymer solution, and Si + PAH-50 mM of both the silicate and polymer (inset shows the optical images of mixtures of PAH + Si at various pH values). (b) Ionization degrees of PAH (see the Experimental Section for the details of calculation and Figure S2). (c) Silicate species abundance (calculated with PHREEQC) at different pH values.

Since polyamines are weak organic bases and silicic acid is a weak inorganic acid, both are sensitive to protonation and deprotonation. Therefore, our investigations focused on the interactions between PAH and silicate solutions under various pH conditions (for simplicity, in this work, we refer to all solution species of silicic acid and their conjugated bases and oligomers as silicates). Each stock solution was first adjusted to the tested pH value by the addition of HCl or NaOH, followed by mixing at different pH values (pH 3–12) to yield a solution of 50 mM PAH and 50 mM silicate. In the absence of the polymer, no immediate precipitation was observed in any of the pH values, even though a gel formed in pH values of 5 to 7 in a time scale of days. Nevertheless, the addition of PAH yielded a pH-dependent precipitation that increased in basic conditions (Figure 1 a). The observed turbidity was quantified using absorbance measurements, showing an increase at pH 8 that continued up to pH 11 (Figure 1 a). Above pH 11, an abrupt reduction in turbidity is observed, and at pH 12, no turbidity is detected (Figure S1).

The observed pH-dependent and polymer-induced turbidity suggests an active role for the polymer that is triggered by basic conditions. We performed titration experiments to measure the deprotonation percentage of the polymer from neutral to basic conditions. This shows that the amine groups undergo gradual deprotonation between pH 6 and 11, and that at pH 9, half of the amines are neutral (Figure 1 b). The dominant silicic acid species at our experimental concentration were calculated using PHREEQC (Figure 1 c), showing an elevation in the abundance of charged silicates above pH 8. These observations show that the observed precipitation in the mixture of PAH and silicate correlates with an increasing percentage of neutral PAH and charged silicates, and that the abrupt reduction in precipitation signifies the completion of these two processes.

**Figure 2:**
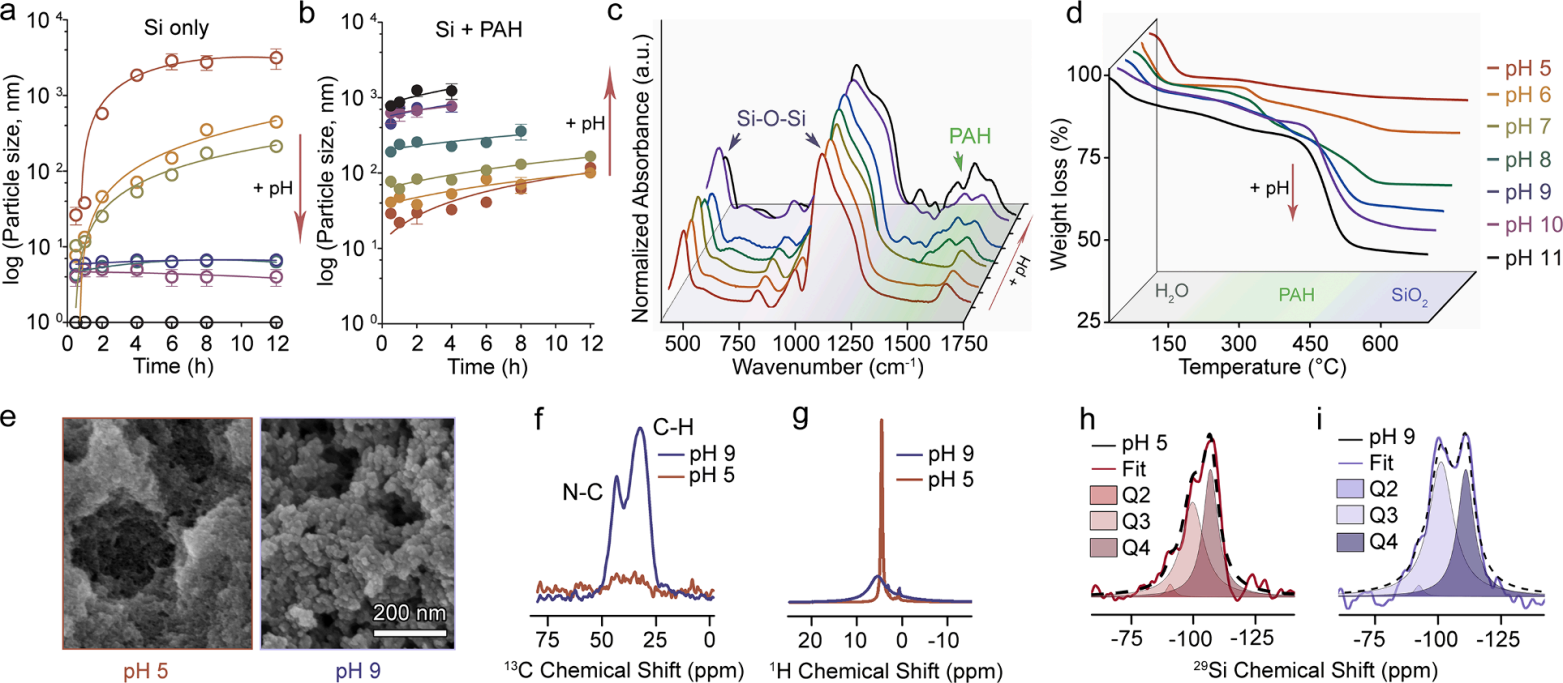
Dynamics and chemical analyses of silica–PAH hybrids formed at different pH values. (a, b) DLS measurements of particle sizes in solutions that contain only silicate (a) or a mixture of silicate and PAH solutions (b). (c) FT-IR spectra and (d) TGA curves of lyophilized precipitates of the Si + PAH system at various pH levels. (e) SEM images of the lyophilized Si + PAH (pH 5,9). (f–i) Solid-state NMR spectra obtained from the lyophilized precipitates of the PAH + Si mixture at pH 5 and pH 9. (f) ^1^H–^13^C cross-polarization MAS NMR spectra. (g) ^1^H MAS NMR. (h,i) ^29^Si MAS NMR spectra were referenced to a Kaolinite set at −92 ppm, and deconvolution plots were obtained according to the fixed values for different *Q* peaks.

We used DLS measurements to investigate the precipitation dynamics in the 50 mM silicate solutions, with and without 50 mM PAH, over the pH range 5 to 11 (Figure 2a,b). At pH 5, the silicate solution without PAH showed steady growth of particles in the first hours, as expected from a sol–gel process.^1,5,32^ As the pH of the silicate solution increases and the saturation level decreases, the gelation rate slows until no precipitation was observed at pH 8 and 9. However, the presence of PAH introduced a remarkable shift in the kinetics relative to the pure silicate system. In acidic conditions (pH 5), the growth of particles slowed down relative to the pure Si system, an effect that is observed up to pH 7. In basic conditions, the influence of PAH is reversed as particles are detected directly after mixing, and their size only mildly grows. This suggests that PAH stabilizes gelation when it is charged at acidic pH values and promotes precipitation in its neutral state via a mechanism that is substantially faster than sol–gel precipitation.

Macroscopically, there is a difference between the clear silica gel, which forms under acidic conditions in the presence or absence of PAH, and the solid-like precipitates that form under basic conditions in the presence of PAH and sediment with time (Figure S3). FTIR spectroscopy showed a gradual increase in the relative intensity of the PAH vibration with higher pH values, demonstrating the presence of the polymer in the silica precipitate (Figure 2c, Figure S4). This higher PAH content in precipitates that form under basic conditions was corroborated by thermal gravimetric analysis (TGA). These analyses show increased weight loss from ∼300 °C due to the occluded organic content within the silica precipitates (Figure 2d). Nevertheless, scanning electron microscopy (SEM) images show comparable morphologies of dried granular materials obtained from pH 5 and 9, with a somewhat larger granule size at pH 9 (Figure 2e).

Solid-state nuclear magnetic resonance (ss-NMR) spectroscopy was used to elucidate the chemical differences between precipitates that formed in the presence of PAH at pH 5 and 9. The precipitates that formed at pH 9 had a much higher PAH content (indicated by the intensity of the ^13^C NMR signal), while the sample at pH 5 had a higher water content (indicated by the sharpness of the ^1^H NMR water signal at 4.9 ppm (Figure 2f,g)). The ^29^Si MAS NMR spectra acquired by direct excitation were used to quantify the degree of silica polymerization. Both samples show resonances centered at −110, – 100, and −90 ppm, which can be assigned to Q4, Q3, and Q2 species, respectively (Q4 being a fully saturated Si–O–Si network and Q0 being monomeric Si(OH)_4_) (Figure 2h,i). Deconvolution and integration of the different sites showed a higher Q3/Q4 ratio in basic conditions (a ratio of 1.7 at pH 9 vs 1.1 at pH 5, Figure 2h,i, Figure S5). These observations suggest that the PAH-induced precipitates at basic pH are a hybrid composite that is PAH-rich, less hydrated, and less condensed than the silica gel that forms under acidic conditions. The higher hydration level of the silica gel can be the result of its architecture, which traps water molecules but is not chemically linked to polymer molecules. This points to a unique effect of the polymer on silica precipitation that is different from the simple catalysis of the polymerization reaction.

**Figure 3:**
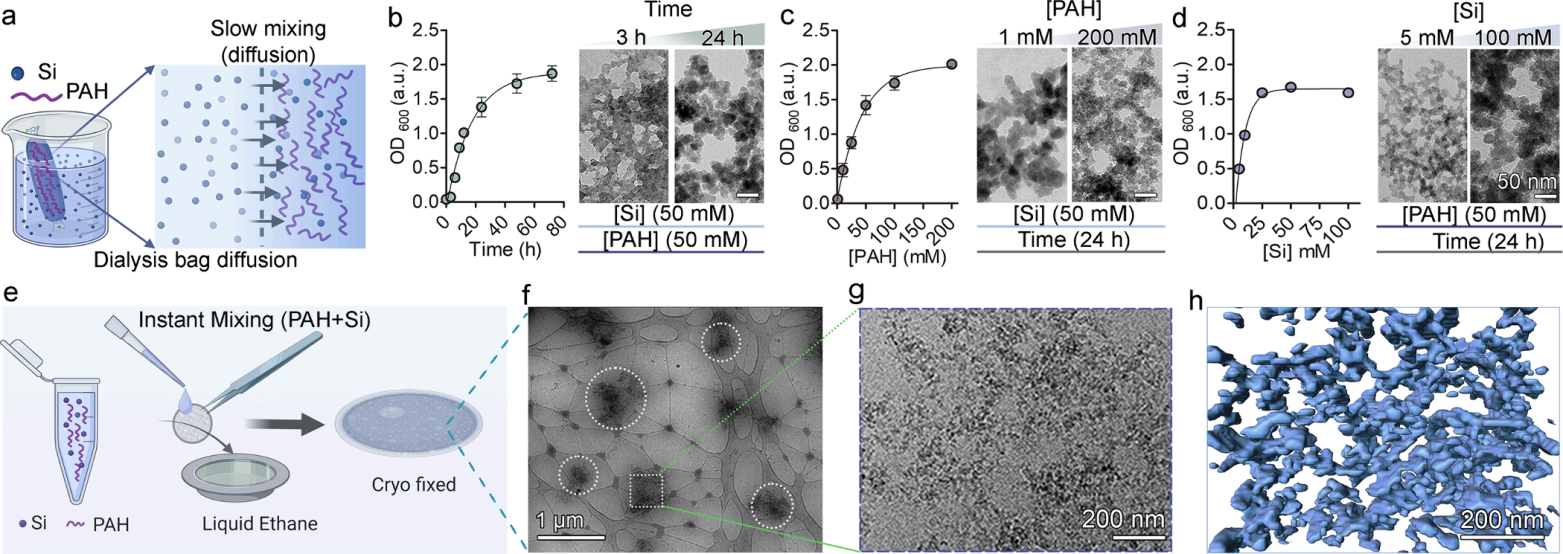
PAH-mediated silicification is different from particle nucleation and growth. (a) Schematic of the experimental setup for slow diffusion of silicate into PAH solution. (b–d) Changing time (b), PAH concentration (c), and silicate concentration (d) in the diffusion experiments. For each variable, turbidity evolution is shown, as well as characteristic TEM images of the samples at two conditions, demonstrating a conserved nanoparticular morphology. (e–h) cryoEM images of the PAH + Si system at pH 9. (e) Schematic of cryofixing of a PAH + Si mixture. (f) Low-magnification cryoEM image showing micrometer-scale aggregates (white circles). Note that the lacey pattern is only the support for the vitreous sample. (g) Higher-magnification image of an aggregate demonstrating a nanoscopic granular silica network. (h) 3D volume rendering of tomography data of a representative aggregate.

To investigate the underlying chemical process, we tested the hypothesis that the observed precipitates could grow larger if the kinetics of the process are slowed down. This was inspired by previous studies showing that amine-containing macromolecules can induce the formation of micron-sized spheres.^33^ The adjusted experimental setup consisted of a dialysis bag containing 50 mM PAH, which was placed in a 50 mM silicate solution, allowing for slow diffusion of silicate into the bag (Figure 3 a). Indeed, the reaction was slower, with turbidity gradually increasing over time, before reaching saturation by 24 h (Figure 3 b). However, transmission electron microscopy (TEM) images showed a nanometer-sized particle network that changed only slightly with extended diffusion time (Figure 3 b and Figure S6). We varied the PAH concentration to test if a lower polymer concentration would lead to a slower catalysis that would allow the particles to grow with time, but also here, the precipitates had a constant size of the particles with turbidity values that correlated with the PAH concentration (Figure 3c and Figure S7). Similarly, changing the silicate concentration did not result in particles larger than a few tens of nanometers (Figure 3d and Figures S8 and S9). The fact that the silica precipitates did not grow in size at any of these conditions points to a precipitation process that differs from particle growth by the addition of soluble building blocks.

We explored the native-state morphological features of the precipitates using cryo-electron microscopy (cryoEM). Samples of the precipitates were vitrified and imaged under their hydrated native conditions (Figure 3e–g). Low-magnification images show micrometer-sized aggregates that contain dense precipitates (Figure 3f). This is in line with the DLS data (Figure 2b) that detect large aggregates and not distinct nanometer-sized particles. A 3D volume reconstruction at higher magnification showed that the aggregates are interconnected nanoscale networks (Figure 3g,h). Altogether, these observations point to a precipitation mechanism that is not emerging from the distinct nucleation events of particles that grow. In classical nucleation and growth, each particle is independent and has a size that grows with the reaction, whereas in this case, there are no distinct particles but only micrometer-sized networks that increase in quantity.

The involvement of such nonclassical precipitation regime is also evident by its induction at unusually high pH values. To evaluate the saturation state of the 50 mM silicate solutions, we followed the sizes of silica particles with DLS in the absence of a polymer. Below pH 8, these particles grew with time, indicating supersaturation. However, above pH 8, we observed the dissolution of these particles, indicating exceeding undersaturation in more basic conditions (Figure S10). Therefore, the PAH-induced precipitation is favored in “nominally” undersaturated silicate solutions. Clearly, precipitation is thermodynamically impossible in undersaturated environments, necessitating a further exploration for an explanation for this unusual chemical regime.

**Figure 4:**
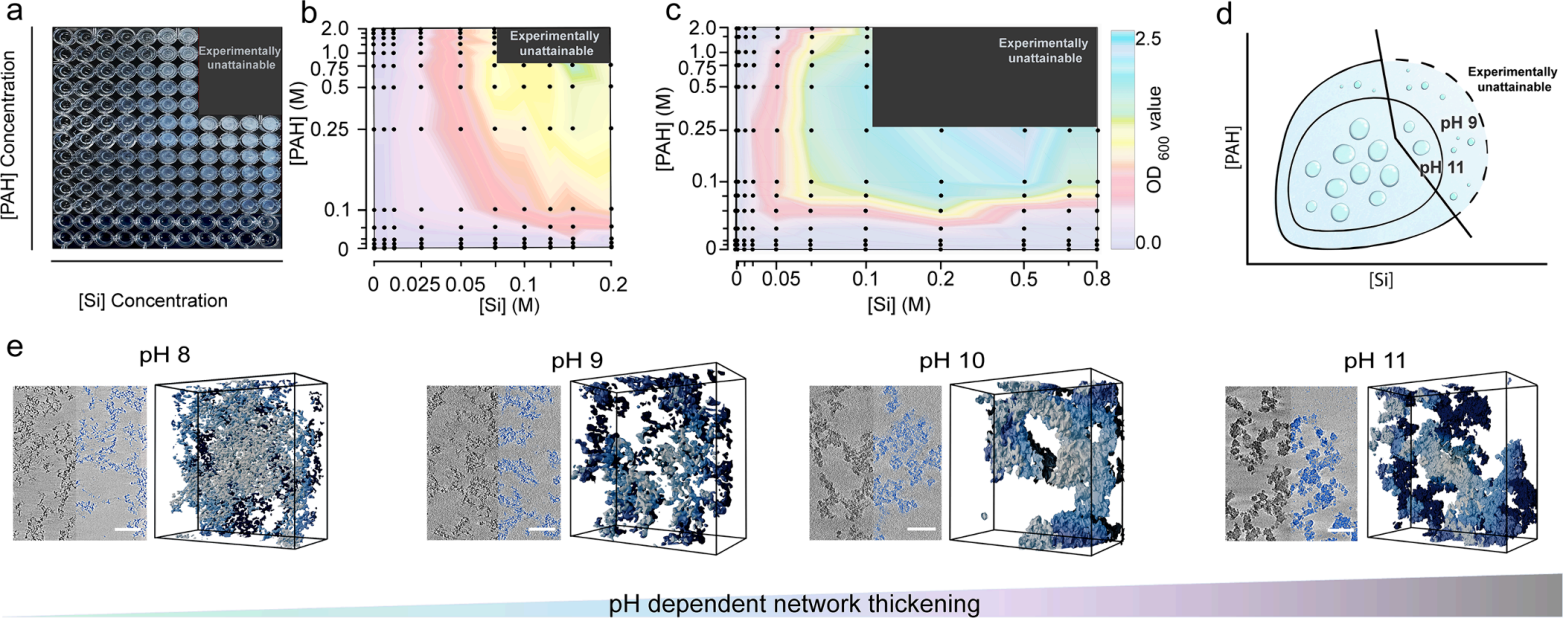
Associative phase separation in the Si + PAH system. (a) Photographs of well plates with various mixtures of Si and PAH at pH 9. (b) Contour plot of turbidity measurements of the same samples. (c) Turbidity contour plot at pH 11. Turbidity values were plotted in contour diagrams, taking the offset reciprocal scale in both axes. (d) Schematic phase diagrams for the PAH–Si system. (e) Cryo-ET of 50 mM PAH and Si at several pH values. For each experiment, a tomographic slice is shown next to a corresponding volume segmentation of the dense silica network (scale bars 100 nm), showing how the network thickens with higher pH.

The behavior of the PAH–silicate system at basic pH values, namely, instantaneous precipitation that yields micron-scale structures, is reminiscent of an associative phase separation of two immiscible solutions.^34−36^ To test if a phase separation regime matches the experimental system, we established a phase diagram by mixing solutions with varying compositions of up to 2 M PAH and 200 mM silicate at pH 9. These maximal values are limited by the solubility of the polymer and the gelation of the silicate. A matrix containing various compositions in this parameter space was prepared in multiwell plates. Visual inspection of these solutions showed the appearance of turbidity when precipitates formed (Figure 4a), which was not sensitive to the salt content in the solutions (Figure S11). Turbidity measurements of each well are presented as a contour plot similar to a phase diagram (Figure 4b). Indeed, the properties of this “phase diagram” are characteristic of an associative phase separation, where demixing occurs in a specific metastable region, and the system is stable in other compositions. Because the physical limitation on concentrations makes only a small fraction of the phase diagram accessible, we conducted further investigation at pH 11, allowing us to reach 800 mM silicate. These conditions show a larger fraction of an unstable region and emphasize that the system behaves as expected for associative phase separation and differently from precipitation induced by a concentration product that should show a monotonous hyperbolic relation (Figure 4d).

We sought an experimental observation that would relate the phase separation process to the structural properties of the precipitates. Since the PAH-induced precipitation is pH-dependent, different pH values were expected to yield different compositions of the dense phases, resulting in different morphologies of the final hybrid material. Indeed, cryo-electron tomography (cryo-ET) of samples prepared at different pH values shows that the phase-separated network of silica hybrids has different characteristic sizes that increase with increasing pH (Figure 4 e, Figures S12 and S13). This structural trait further supports associative phase separation as the initial step of this reaction.

**Figure 5:**
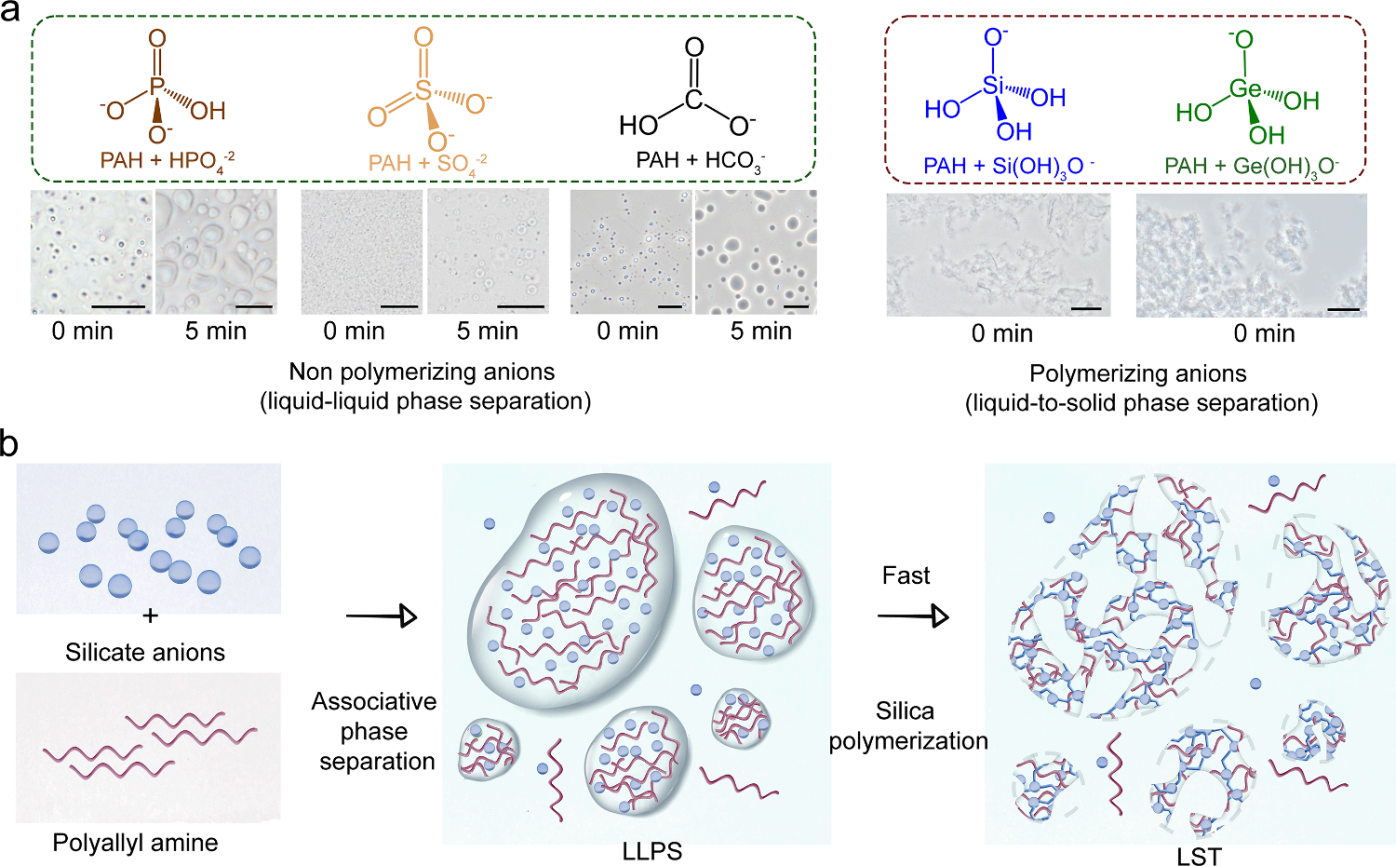
Two-step phase separation model for oxyanions and PAH. (a) Optical images of liquid–liquid phase separation of PAH with five oxyanions at pH 9 and the coalescence with time in the case of phosphate, sulfate, and carbonate (scale bars 20 μm). (b) Model of the two-step mechanism, where first the PAH and Si system phase separate to form dense droplets, which is followed by liquid-to-solid (LST) silica polymerization within the droplets to form interconnected structures.

We further explored the origin of the observed phase separation by replacing silicate with other oxyanions under similar conditions. Upon mixing 50 mM PAH with 50 mM sodium germanate (Ge(OH)_3_O^–^), phosphate (HPO_4_^–2^), bicarbonate (HCO_3_^–^), or sulfate (SO_4_^–2^) at pH 9, turbidity appeared (Figure 5a). This conserved behavior of PAH with various anions suggests nonspecific interactions that drive these phase separation processes. Notably, the dense phase from the nonpolymerizing oxyanions (phosphate, bicarbonate, and sulfate) behaves like a liquid phase that coalesces into bigger droplets. In contrast, the silicate and germanate further polymerize in the dense droplets to form solid networks (Figure 5a). This polymerization of silicate and germanate is a secondary liquid-to-solid phase transition.

In view of the reported results, it is not surprising why so many functional roles were attributed to polyamines in silicification.^9^ We show that even a specific polyamine, PAH, has disparate, pH-dependent effects on silicate solutions. The unexpected side of this work is the identification of the PAH-mediated formation of silica networks at high pH values. The PAH–silica hybrid is distinct from the inorganic silica that forms via the sol–gel route (for example, the reduced water content might be the manifestation of the dense liquid from which the solid hybrid forms, Figure 2g), and its formation process is driven by other chemical forces.^1,3^ The PAH-mediated precipitation is very fast and yields micrometer-sized droplets, which is controlled by the protonation state of the components rather than by silica supersaturation.

We propose that this process is initiated by the immiscibility of the polymer and silicate solutions, which leads to associative liquid–liquid phase separation, yielding dense and supersaturated PAH–Si droplets (Figure 5b). At basic pH conditions, an associative liquid–liquid phase separation drives the formation of polymer- and silicate-rich droplets. Because weak intermolecular forces drive this process, supersaturated condensates can form even when the mother solution is undersaturated.^37−40^ This supersaturation leads to a second phase separation where a network of silicate-rich phases solidifies rapidly. The fact that this secondary phase separation gives rise to a solid silica hybrid prevents spatial rearrangement and droplet coalescence, as can be seen with liquid droplets.

In the current experiments, we could not directly detect the dense liquid droplets before the onset of silicification. Even cryo-EM samples that were vitrified as fast as feasible (only a few seconds after mixing the solutions) had solid silica networks within the micrometer-sized droplets (Figure S13). The liquid–liquid phase separation (LLPS) is thus supported indirectly by the similarity to other oxyanions (Figure 5a) and directly by the fast formation of droplets (Figure 2b). Nevertheless, the observation that different pH values, and hence different driving forces for LLPS, affect the silica hybrid morphology (Figure 4e) is in accordance with other systems where solidification follows an LLPS step.^31,41−43^ Thus, it is an attractive option to use LLPS principles as regulatory handles for the formation of silica hybrids with convoluted shapes.^44^

The elucidation of this LLPS-driven silicification process opens various avenues for designing new functional materials. First, this process uses only biocompatible precursors in an aqueous environment and mild conditions, which are prerequisites for sustainable materials. We demonstrated that system parameters, such as pH, are translated to material properties, such as porosity. This can be advantageous for two opposite approaches. First, changing the reaction parameters can be used as flexible handles to design a product, and second, when a specific product is desired, the vast realm of polymer properties can be mined to find the right polymer properties to achieve the desired outcome. Such organic–inorganic materials are promising in drug delivery applications as various active biomacromolecules can be embedded and stabilized by the silica while preserving activity and benefiting from the slow dissolution of silica.^7,45^ On the other hand, these materials are also advantageous for catalysis due to their inherently high surface area and the possibility of controlling their structure and composition merely by changing the reacting ingredients. As so many novel materials emerged from the conventional sol–gel processes,^2,3,32^ the LLPS route might provide significant addition to the material portfolio.

Even though the use of polyamines for silicification is inspired by observations in organisms,^5,6,46^ it does not mean that this process represents biosilicification. The main difference is that biogenic silica is space-filling and not porous. Nevertheless, the concept of polymer-induced silicification in dense environments should be considered in vivo. Since the cellular environment is crowded with various macromolecules, it will be interesting to investigate how the polyamines affect silicification when they are in a dense phase prior to reaction with silica.^28^ It is also important to widen bioinspired studies beyond the neutral to mildly acidic pH range as we demonstrate that charged silicates interact differently from the neutral silicic acid with polyamines.

## Conclusions

We establish a role for PAH as a driver of phase separation in undersaturated silicate solutions. This reaction is driven not by supersaturation but by weak interactions between the components, dictated by their protonation state. It leads to the formation of dense and supersaturated droplets in which a solid silica–PAH hybrid forms in a network structure. This material is different, both in its properties and in its formation process, from the well-known silica sol–gel, therefore opening new directions to design silica materials and to continue and elucidate the biological processes that endow spectacular regulation over silica formation for many types of organisms.

## Experimental Section

### Materials and Methods

Poly(allylamine hydrochloride) (PAH·HCl, 17.5 kDa), sodium silicate solution (Si, ((NaOH)_*x*_(Na_2_SiO_3_)_*y*_·*z*H_2_O, 27% SiO_2_)), Na_2_HPO_4_, NaHCO_3_, Na_2_SO_4_, and GeO_2_ were purchased from Sigma-Aldrich. Ultrapure water from the Milli-Q IQ 7003 Ultrapure Lab Water System (Merck) was used for solution preparation.

### Silica Precipitation

100 mM polyallylamine (PAH) and silicate/silicic acid (Si) were prepared by dissolving PAH·HCl in Milli-Q water and diluting the sodium silicate solution. The initial pH was adjusted with HCl/NaOH. The two solutions were mixed with the same volume ratio. Silica precipitation was instantaneous in basic conditions, and slow gelation was found in acidic or neutral conditions. The diffusion-controlled silica precipitation reaction was performed in a dialysis bag setup experiment. Polymer solutions of different concentrations at pH 9 were taken in dialysis tubing (cellulose membrane molecular, weight cutoff = 14 000 Da) and placed into different silicate solutions of pH 9. The precipitates were collected by centrifugation (15,000 rpm, 5 min), washed thrice with Milli-Q water, and lyophilized for dry state measurements and studies. Turbidity was measured in a 96-well plate of 0.1 mL of solution using a Tecan plate reader (Infinite m 200) at 600 nm wavelength.

### Dynamic Light Scattering

DLS experiments were conducted using a Zetasizer Nano ZSP (Malvern Instruments, United Kingdom) equipped with a 633 nm laser to measure particle sizes in solution. Particle sizes were determined by the intensity distribution and presented as the average values of three replicated measurements.

### Polymer Titration

Polymer titration was performed with a pH meter (Eutec pH 700) by setting up a titration experiment. The titrate solution PAH·HCl (50 mM, 10 mL) having an initial pH of 2 was prepared and titrated with NaOH. pH values were measured with stepwise addition (5 μL) of NaOH (2.5 M). The exact titration points were resolved from the first-order derivative of the titration curve. The first titration point indicates the starting of the deprotonation of PAH·HCl where we assume the starting of deprotonation following the second titration point with 100% deprotonation of the polymer solution. The p*K*_a_ and % of deprotonation were calculated following the equations
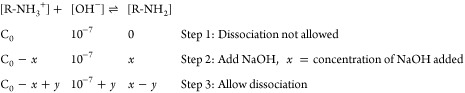

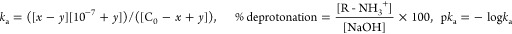


### Fourier Transfer Infrared Spectroscopy

Lyophilized samples were prepared for IR spectroscopy on a KBr plate using a hydraulic palletizer. IR spectra were collected using a Nicolet Summit X FTIR Spectrometer (Thermo Scientific).

### Thermogravimetric Analysis

Lyophilized samples were subjected to thermal gravimetric analysis with an SDT Q600, TA Instruments, USA. Experiments were conducted in an oxidative environment under a constant airflow (100 mL/min) with a heating rate of 10 K/min. When the temperature approached 700 °C, we considered that all of the organic parts were combusted, and the Si contents were measured.

### Scanning Electron Microscopy

Lyophilized samples were mounted on carbon tape attached to aluminum SEM stubs and coated with 4 nm iridium using a compact coating unit (CCU-010, Safematic). The images were acquired in a scanning electron microscope (Sigma 500 SEM, Zeiss) equipped with an in-lens detector (W.D. 2.5 mm) under 4 kV.

### Solid-State NMR

Solid-state NMR experiments were performed on a 9.4 T Bruker Avance III. The ^29^Si spectra were referenced to a Kaolinite set at −92 ppm, and the ^1^H and ^13^C spectra were referenced to adamantane at 1.8 and 38.5 ppm (for the CH resonance). All experiments were acquired using a 4.0 mm double resonance probe, at room temperature, and a MAS rate of 12.5 kHz. The ^1^H MAS NMR spectra were collected using single pulse excitation with a recycle delay of 1 and 4 scans. ^1^H–^13^C cross-polarization experiments were performed using a high-power decoupling of 70 kHz, recycle delay of 3 s, 1024 scan, and contact time of 0.5 ms. The ^29^Si MAS NMR spectra were acquired with a high-power decoupling of 70 kHz, recycle delay of 300 s, and 144 scans. Quantification was done by integrating the resonances using MATLAB.

### Transmission Electron Microscopy

A 2 μL aliquot of sample solution was drop-casted on a carbon-coated Cu TEM grid (200 mesh), and the solution was blotted on filter paper. The grids were dried and stored under a desiccator under a vacuum. TEM images were acquired with an FEI Tecnai T12 TEM microscope (Thermo Fisher Scientific, USA) equipped with an XF416 TVIP camera (TVIPS GmbH, Germany) with an accelerating voltage of 120 kV.

### Cryo-TEM and Tomography

Sampling for cryo-TEM was done by diluting 10 times with the supernatant of the solution. The sample solution was vitrified on the lacy carbon grid (200 mesh copper). The grids were plasma cleaned, and a 2 μL diluted aliquot was drop-casted on the carbon side of a grid with a 2 μL supernatant wetting solution on the copper side. Grids were blotted for 2 s and vitrified in liquid ethane using a Leica EM GP automatic plunger (Leica, Vienna, Austria) at 25 °C under 90% humidity conditions. The vitrified specimens were stored in liquid nitrogen until use. Cryo-TEM imaging and tomogram data collection was performed on a Talos Artica (Thermo scientific) with a Falcon 4i camera with an accelerating voltage of 200 kV. The tilt series data were collected in counting mode at a magnification of 45000×, pixel size of 0.31 nm, using the dose-symmetric method with 2° increments over a −60 to +60° angle. The images were acquired with an objective aperture of 30 μm at a defocus range of 5–8 μm, and the total dose was maintained from 120 to 160 e–/Å^2^. The tilt series data were reconstructed with the patch tracking method with IMOD software. The reconstructed data were segmented using Amira 3D software v2021.2 (Thermo Fisher Scientific, Waltham, MA, USA).

### Light Microscope Imaging

Optical images were taken by using a light microscope (Nikon Eclipse Ni-U). The PAH (100 mM) and sodium salt of anions (100 mM) solutions were mixed with the same volume ratio at pH 9 and drop-casted on a glass slide, and images were captured.

## Supporting Information Available

DLS, turbidity, and titration measurements of the Si–PAH solution; detailed FTIR, NMR, TEM, and cryo-TEM analyses of the precipitate sample (PDF)

## ABBREVIATIONS

PAH: polyallylamine
Si: silicic acid/silicate
