# Supplementary material for: A pH-Dependent Phase Separation Drives Polyamine-Mediated Silicification from Undersaturated Solutions
Source: ACS Nano. 2024 Dec 6;18(50):33998–4006. doi: 10.1021/acsnano.4c08707 (PMC11656833; doi:10.1021/acsnano.4c08707)
Supplement: Supplementary file 1 — nn4c08707_si_001.pdf [file nn4c08707_si_001.pdf]

Supporting Information

A pH-dependent Phase Separation Drives Polyamine-mediated Silicification from Undersaturated Solutions

Protap Biswas [a], *Nitzan Livni* [b], *Debojit Paul* [a], *Lior Aram* [a], *Razi Safadi* [a], *Neta Varsano* [c], *Nadav Elad* [c], *Roman Kamyshinsky* [c], *Michal Leskes* [b], *Assaf Gal* *[a].

[a] Department of Plant and Environmental Sciences, Weizmann Institute of Science, 7610001 Rehovot, Israel, [b] Department of Molecular Chemistry and Materials Science, Weizmann Institute of Science, 7610001 Rehovot, Israel, [c] Department of Chemical Research Support, Weizmann Institute of Science, 7610001 Rehovot, Israel

Correspondence E-mail: assaf.gal@weizmann.ac.il

Supplementary Figures

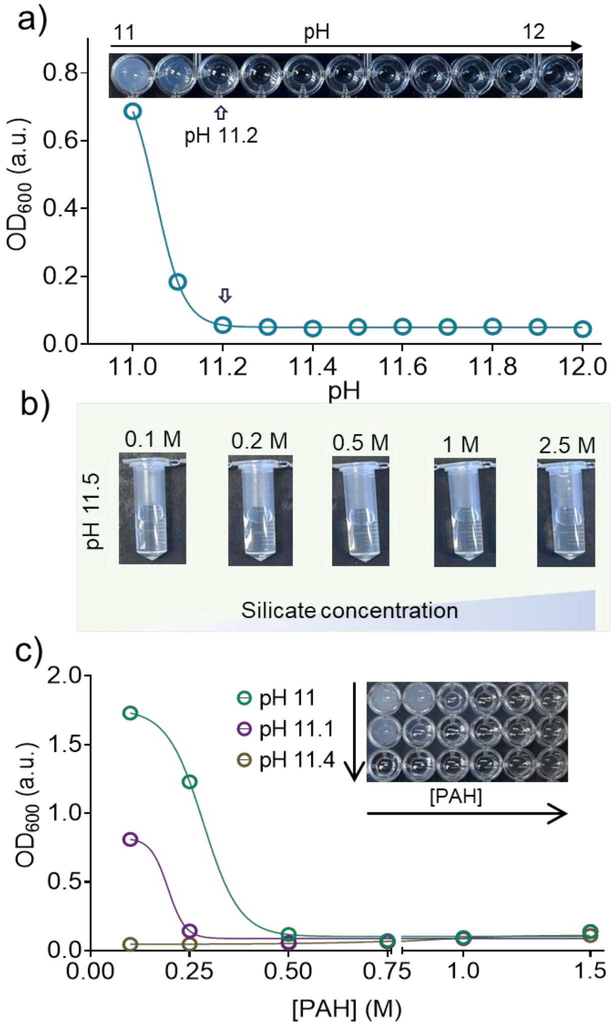

Figure S1: Silica precipitation at pH 11 and above. a) Turbidity measurements under various pH condition from pH 11-12 of 50 mM Si + 50 mM PAH mixtures. Inset shows optical image of the solutions. b) Optical images of the clear solutions of 50 mM PAH and different concentrations of silicate solutions at pH 11.5. This shows that silicate concentration is not the factor that limits precipitation. c) Turbidity measurements of solutions of 50 mM silicate solution and varying PAH concentrations under different pH conditions. This shows that at pH 11.1 some precipitation occurs at high [PAH], but above this pH no precipitation occurs. Taken together, these observations suggest that above pH 11 the abrupt reduction in precipitation is not only a factor of supersaturation but also results from the complete neutralization of the PAH and the opposite effect on the silicates (see Fig. 1 b,c).

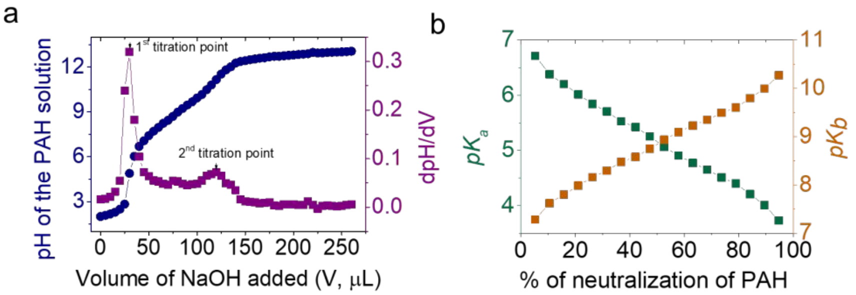

Figure S2: A pH titration curve of PAH (50 mM) with NaOH. a) pH change with the volume of NaOH added and its first derivative. b) calculated *pKa* and *pKb* for PAH.

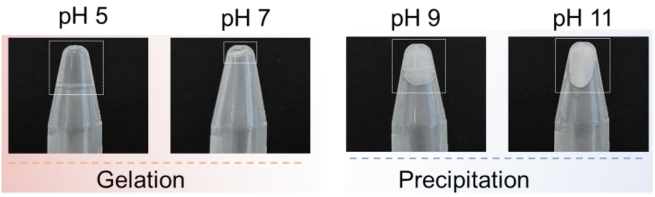

Figure S3: The precipitates obtained from PAH and silicate mixtures at different pH. Acidic and neutral (pH 5, 7) yielded transparent silica gel, while basic pH (9, 11) yielded opaque silica precipitate.

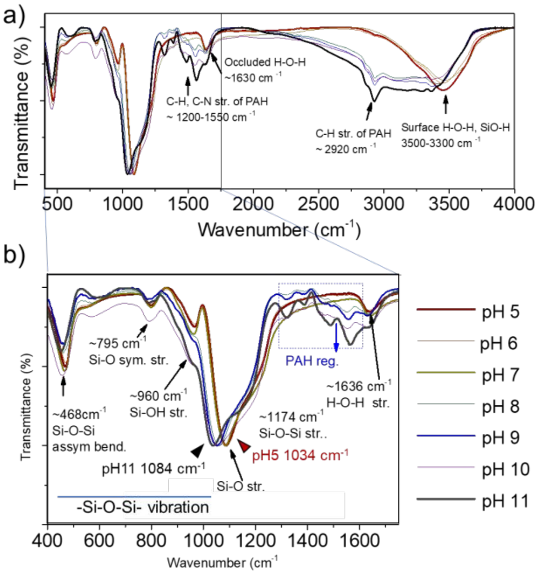

Figure S4: FT-IR spectra of silica precipitates under different pH values in the presence of PAH. a) full spectra. b) Silica and polymer peak analysis.

Si Q peaks	Ratio (pH 5)	Ratio (pH 9)
**Q3**	1	1
**Q4**	0.9	0.6
**Q2**	0.05	0.03

Figure S5: Peak area integration ratios obtained from ^{29}Si NMR.

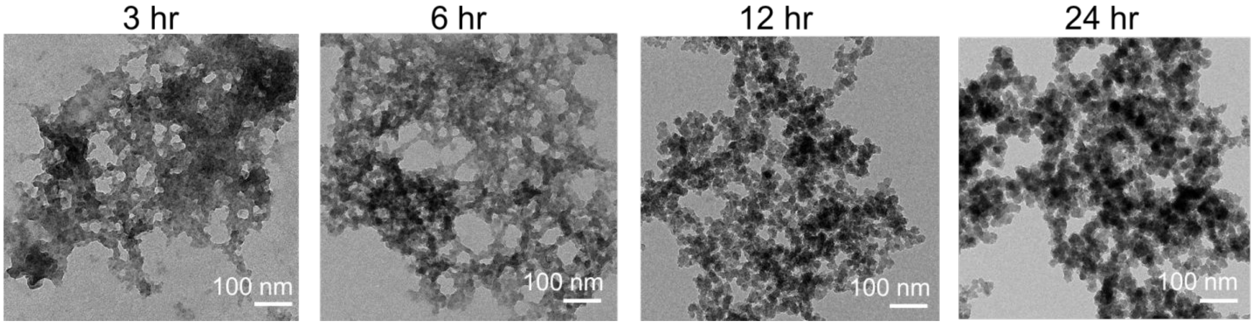

Figure S6: TEM images obtained from dialysis bag experiment from different time interval growth.

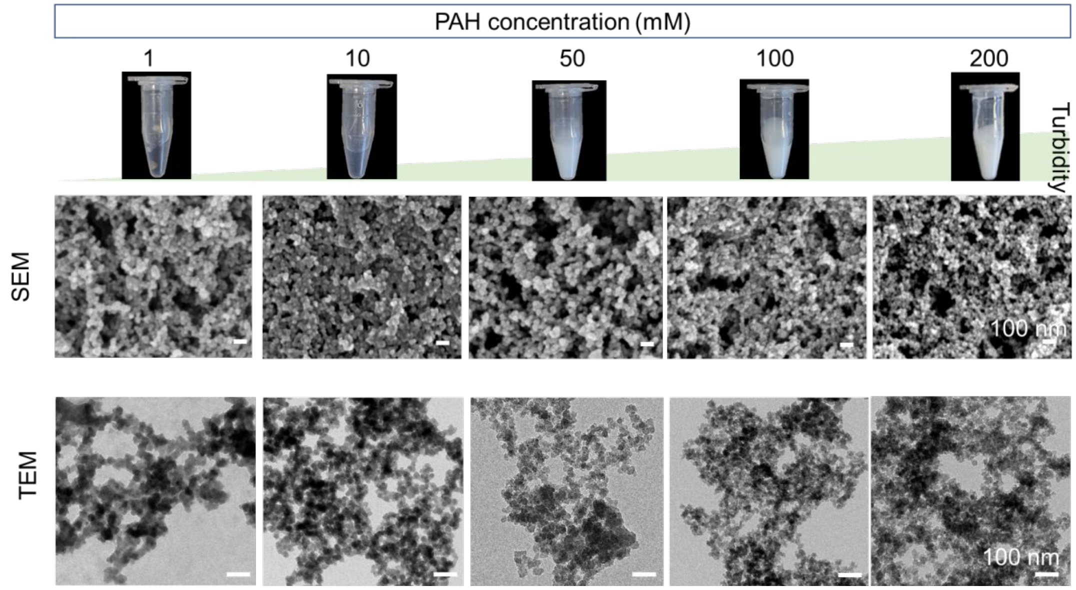

Figure S7: PAH concentration-dependent particle growth in dialysis bag diffusion and corresponding electron microscopic images.

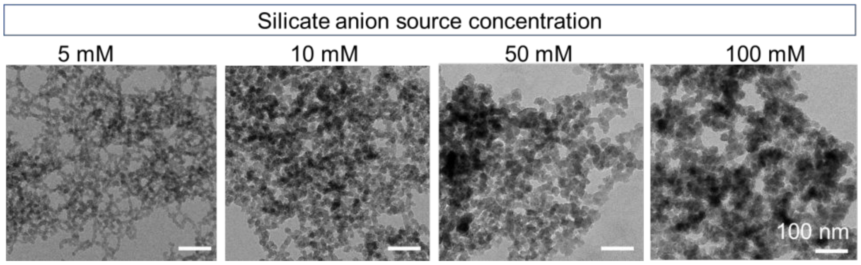

Figure S8: Silicate ion concentration-dependent particle growth in dialysis bag diffusion.

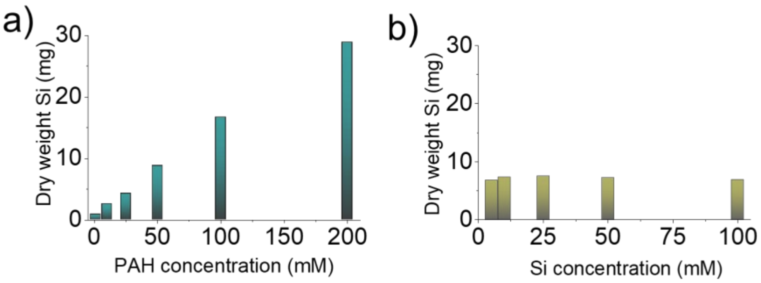

Figure S9: Dry weight of silica precipitates from PAH and Si concentration-dependent experiments in dialysis bag diffusion.

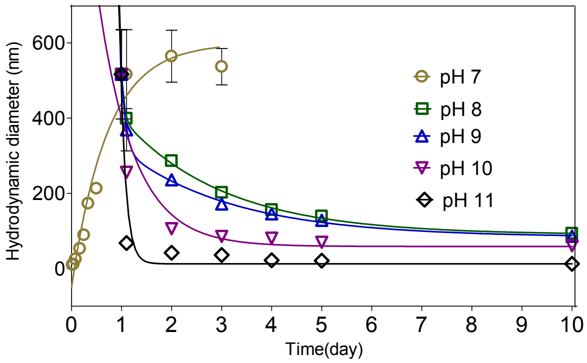

Figure S10: Evaluating the saturation level of 50 mM Si solutions at various pH values. For all experiments, an initial stock of Si solution at pH 7 was aged for 24 hours. After this time, silica particles with sizes of ~400 nm are detected by DLS. The fate of these particles was monitored with DLS after the pH of the system was titrated by the addition of NaOH to various values (pH 8-11). In pH 7, the particles continued to grow until a gel was formed. However, at basic conditions, particle size diminished, indicating particle dissolution. Since dissolution is possible only in undersaturated conditions, we conclude that the system above pH 8 is undersaturated.

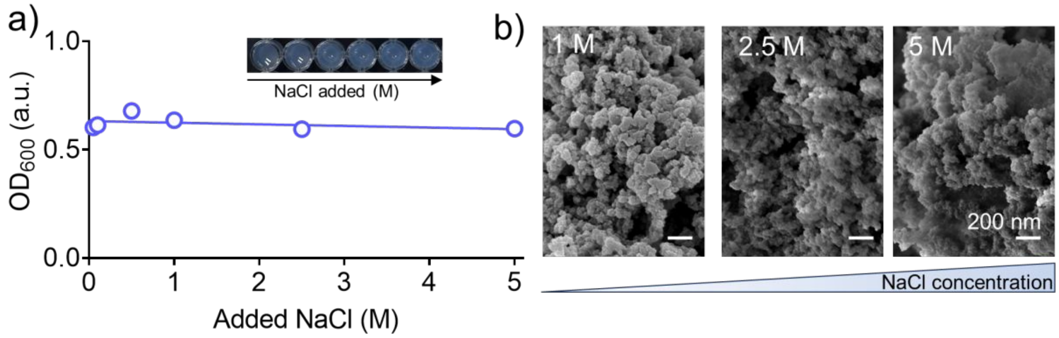

Figure S11: Ionic strength does not affect phase separation-based silica precipitation. a) Turbidity measurements of solutions of 50 mM PAH and 50mM silicate in presence of added NaCl, inset shows optical images of the solutions, b) SEM images of the silica precipitate at different NaCl concentrations.

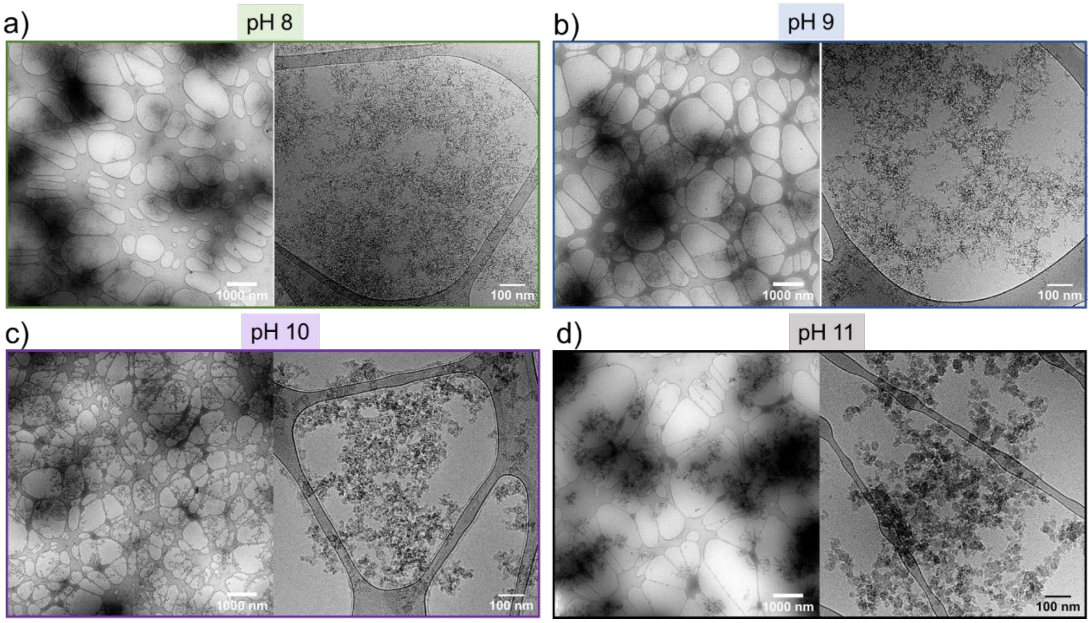

Figure S12: Cryo TEM images at low and high magnifications of the silica aggregates that were formed after mixing PAH and Si at different pH values.

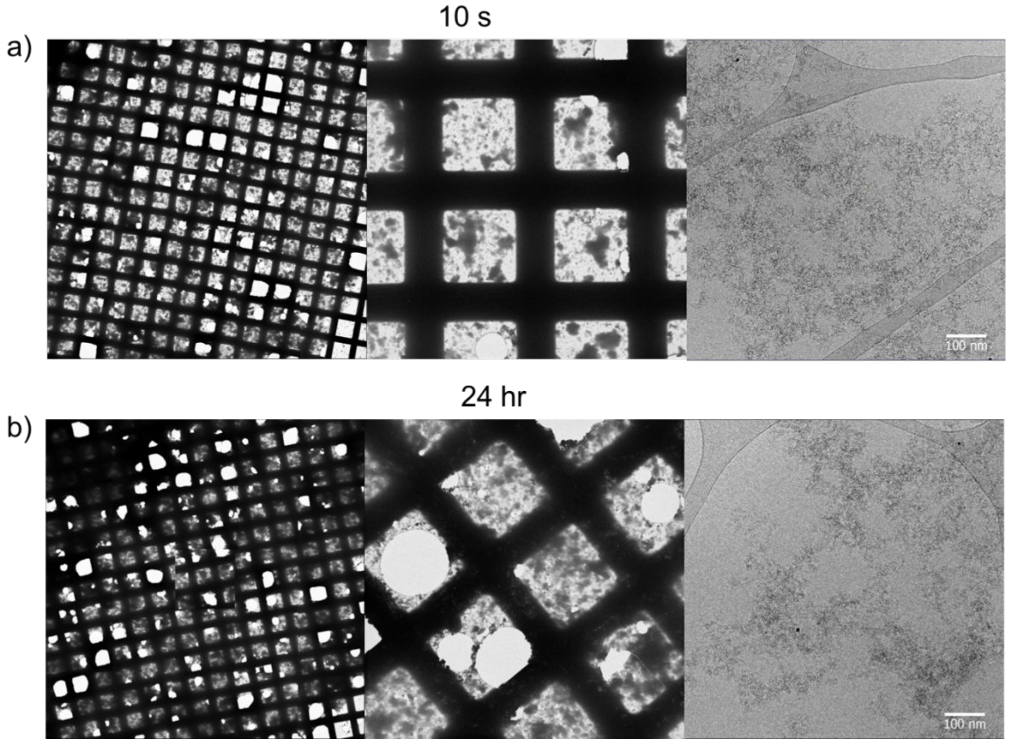

Figure S13: Cryo TEM images of silica precipitates, a) vitrified after 10 s of mixing of PAH and silicate solution, b) samples that were aged for 24 hours and then vitrified. The similarities in the network morphologies suggests that the process reaches a steady state earlier than the fastest vitrification attempt.
